# Contemporaneous Clipping of Unruptured Anterior Cerebral Artery Proximal A1 Segment Aneurysm and Resection of Dural-Based Brain Tumor

**DOI:** 10.7759/cureus.8183

**Published:** 2020-05-18

**Authors:** Zaid Aljuboori, Dale Ding, Brian J Williams

**Affiliations:** 1 Neurological Surgery, University of Louisville School of Medicine, Louisville, USA

**Keywords:** aneurysm, tumor, brain, coexisting, clipping, resection

## Abstract

The coexistence of brain tumors and unruptured intracranial aneurysms is uncommon, so there is limited data regarding management strategies for these cases. Tumor, aneurysm, and patient factors must be considered in the decision-making process. We present a case of a dural-based left temporal brain tumor with an incidental ipsilateral unruptured anterior cerebral artery (ACA) proximal A1 segment aneurysm.

A 56-year-old female presented with progressive headaches and convulsions without focal neurological deficits. Neuroimaging showed a large dural-based left temporal tumor with adjacent vasogenic edema. The patient underwent a cerebral angiography for preoperative tumor embolization, which revealed a small, unruptured intracranial aneurysm arising from the left ACA proximal A1 segment. We performed a left frontotemporal craniotomy for concurrent resection of the dural-based tumor and clipping of the left A1 aneurysm. She elected to proceed, so she underwent a left-sided craniotomy for tumor resection and clipping of the aneurysm. Postoperatively, the patient developed transient, mild right-sided hemiparesis from a left anterior thalamic infarct that resolved before discharge. Follow-up brain magnetic resonance imaging and catheter cerebral angiography showed gross total resection of the tumor and complete aneurysm obliteration, respectively. Patients with dual diagnoses of a brain tumor and intracranial aneurysm can be challenging to manage. When intervention is indicated for each lesion and both can be safely accessed from the same operative approach, contemporaneous surgical treatment of the tumor and aneurysm is reasonable in appropriately selected cases.

## Introduction

The association between brain tumors and intracranial aneurysms has been previously described, with significant variability in the reported coincidence rate. The majority of aneurysms in published reports involved the paraclinoid internal carotid artery (ICA), anterior communicating artery, and middle cerebral artery, and the majority of associated tumors were meningiomas, pituitary adenomas, and gliomas [1-6]. The exact relationship between intracranial neoplasms and aneurysms is unknown, but several mechanisms have been hypothesized, including increased regional blood flow, presence of dysgenetic factors, and tumor-induced injury of the arterial wall [2,4,6].

The coexistence of these distinct clinical entities represents a management dilemma, as aneurysmal subarachnoid hemorrhage incurs a significant risk of neurological morbidity and mortality. Prior reports have documented perioperative aneurysm rupture following brain tumor resection. These findings underscore the importance of formulating a treatment strategy that addresses both lesions [1,3,5]. We describe the management of a patient with a dural-based left temporal brain tumor associated with an unruptured anterior cerebral artery (ACA) proximal A1 segment aneurysm. 

## Case presentation

**Figure 1:**
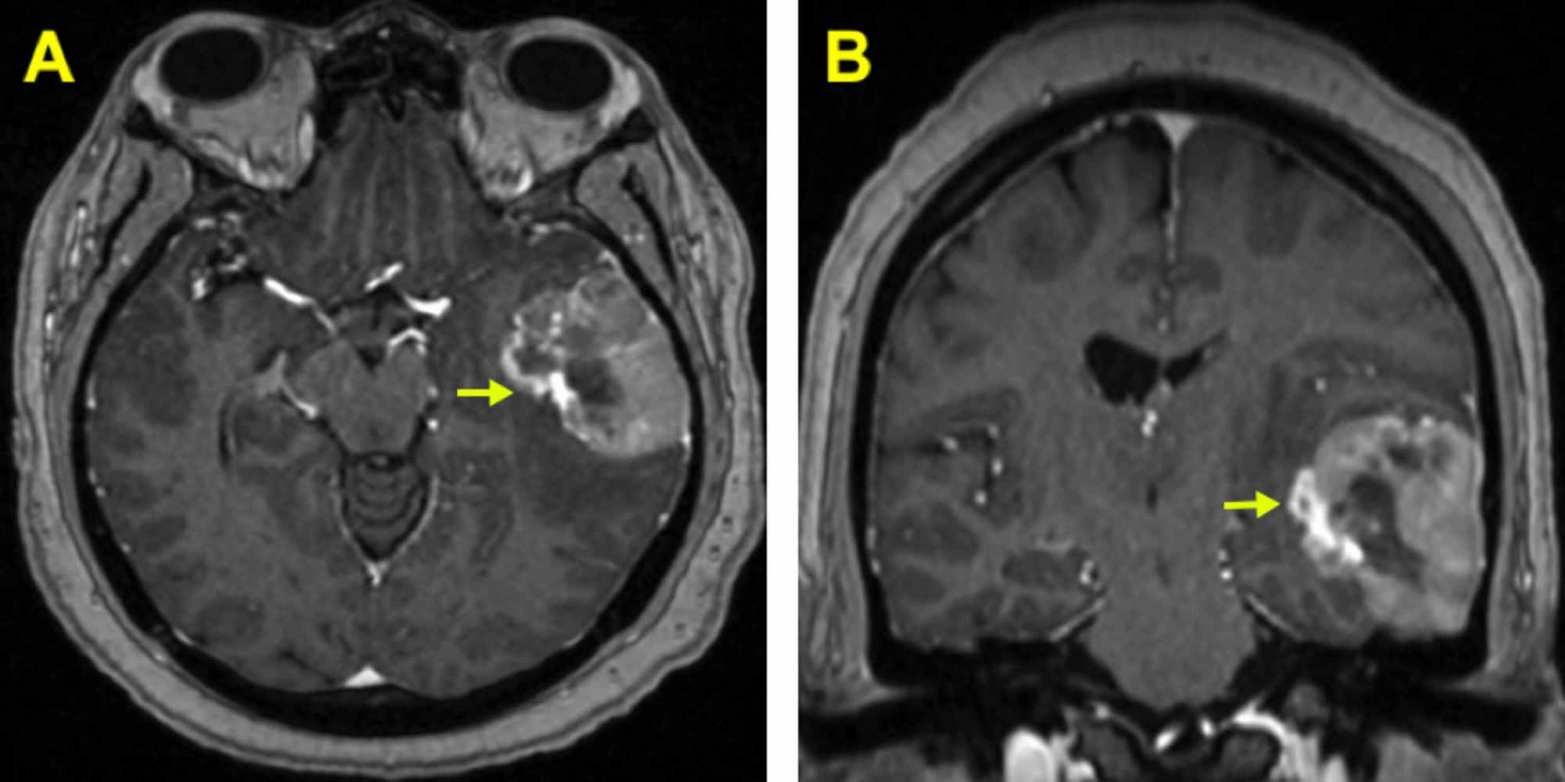
Preoperative MRI brain with contrast shows (A) left-sided temporal lesion (yellow arrow) (axial view), and (B) left-sided temporal lesion (yellow arrow) (coronal view).

A 56-year-old female presented with progressively worsening headaches and occasional convulsions with a normal neurological examination. Neuroimaging showed a large, 5 x 3 cm dural-based left temporal tumor causing locoregional mass effect (Figure 1). Chest, abdomen, and pelvis computed tomography and positron emission tomography showed a small, hypermetabolic lung lesion. The location of the lung lesion precluded both bronchoscopic and needle biopsies. Therefore, we counseled the patient regarding brain tumor resection to obtain a tissue diagnosis and relieve the mass effect. 

**Figure 2:**
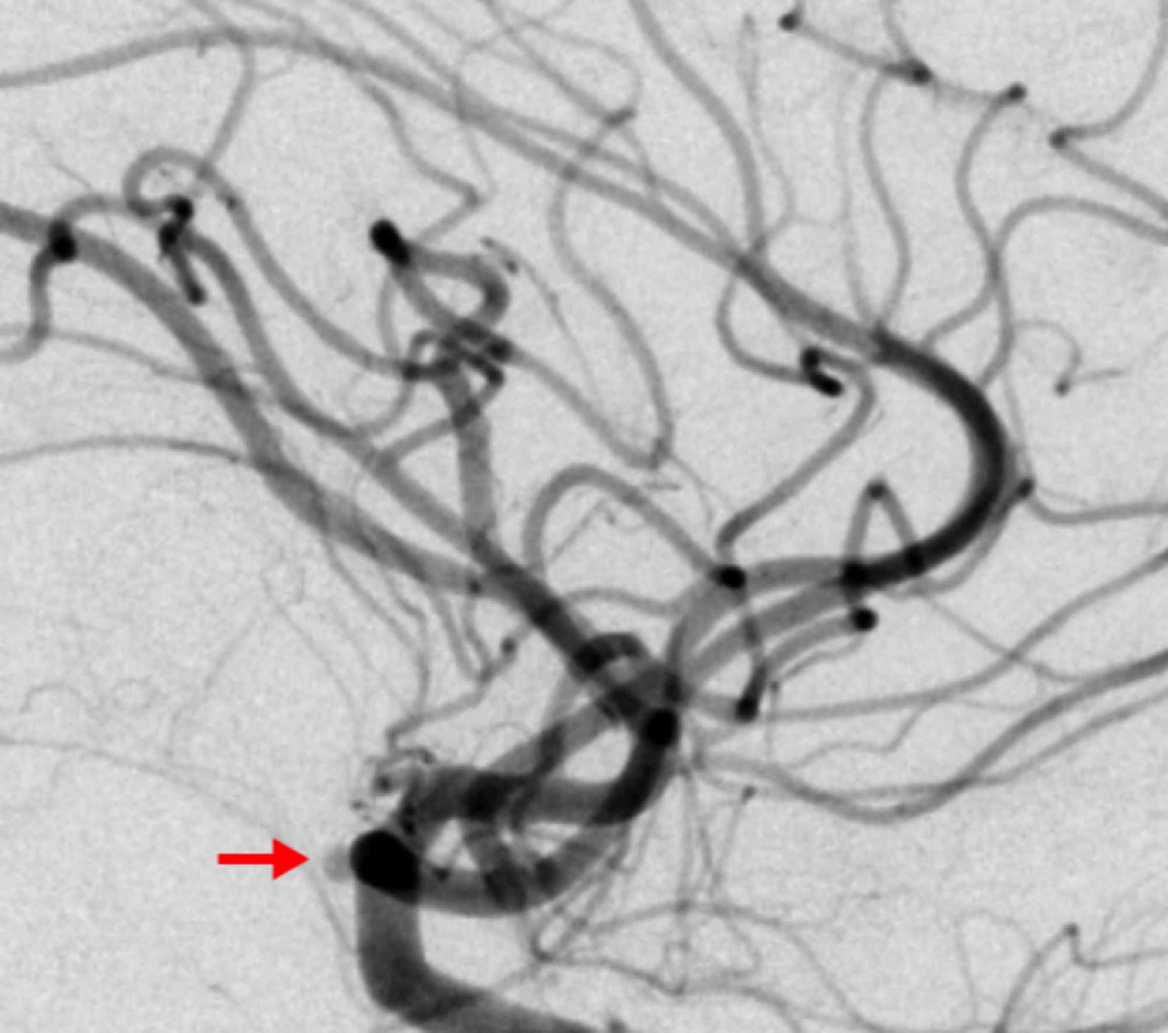
Diagnostic cerebral angiogram (lateral view) of the left internal carotid artery shows the anterior cerebral artery A1 aneurysm (red arrow).

We performed a catheter cerebral angiogram for preoperative embolization of the tumor with n-butyl cyanoacrylate (NBCA), and this incidentally showed a small, 2.0 x 1.6 mm unruptured left ACA proximal A1 segment aneurysm (Figure 2). Given the proximity of the two lesions, we counseled the patient regarding aneurysm clipping during tumor resection, and she elected to proceed with both procedures concurrently. We performed a left frontotemporal craniotomy representing a pterional craniotomy with a posterior extension, including drilling of the lesser wing of the sphenoid bone to the meningo-orbital band, to provide access to both lesions. The dural-based tumor was resected first. We then opened the Sylvian fissure and dissected along the subfrontal corridor to expose the left supraclinoid ICA, middle cerebral artery M1 segment, and ACA A1 segment. The aneurysm was identified arising from the posterior wall of the left A1, immediately distal to its origin from the ICA bifurcation. The adjacent ICA bifurcation perforators were dissected off the aneurysm neck, and the aneurysm was occluded using a single miniature aneurysm clip. 

**Figure 3:**
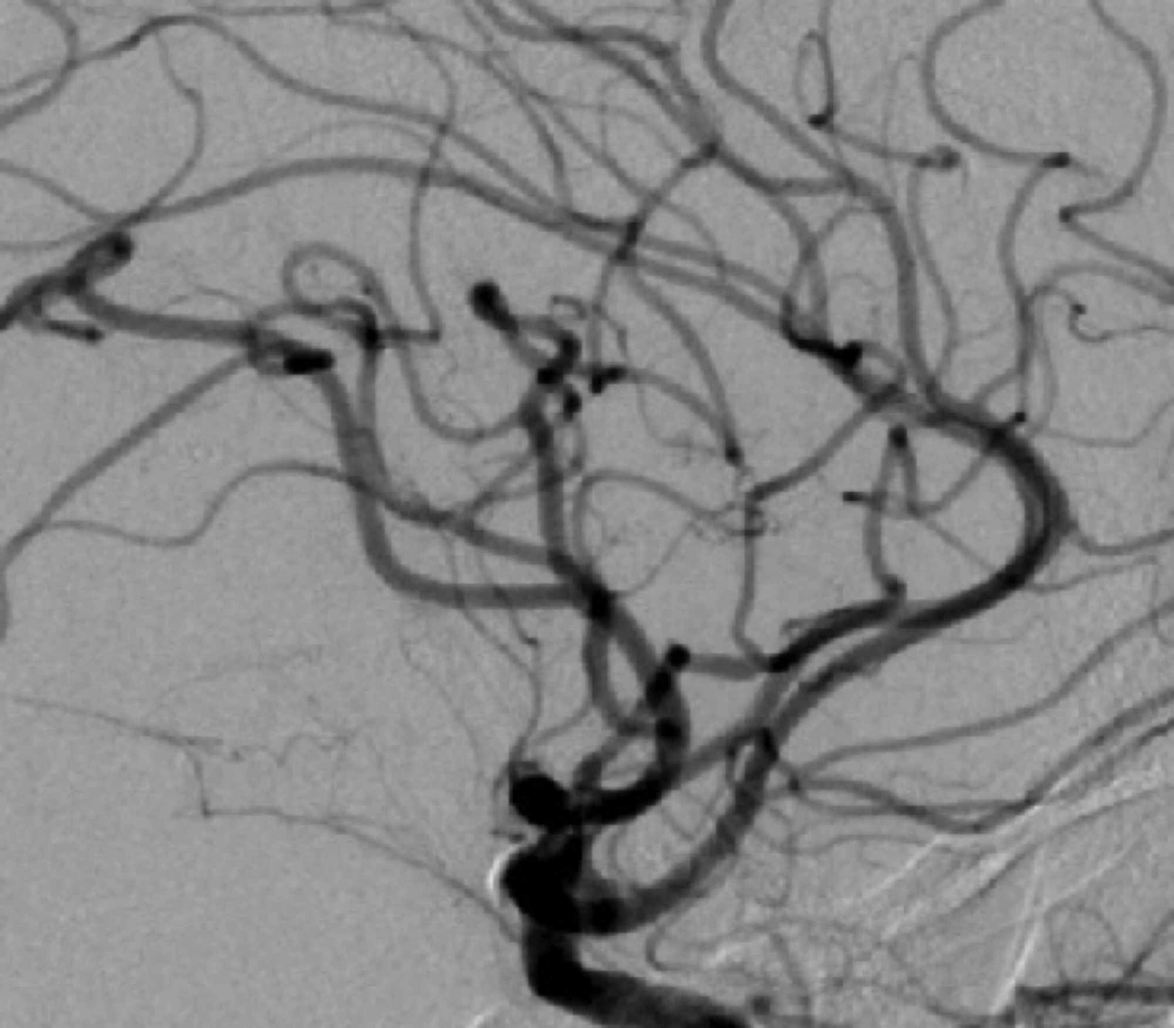
Diagnostic cerebral angiogram (lateral view) of the left internal carotid artery shows complete obliteration of anterior cerebral artery A1 aneurysm post clipping.

**Figure 4:**
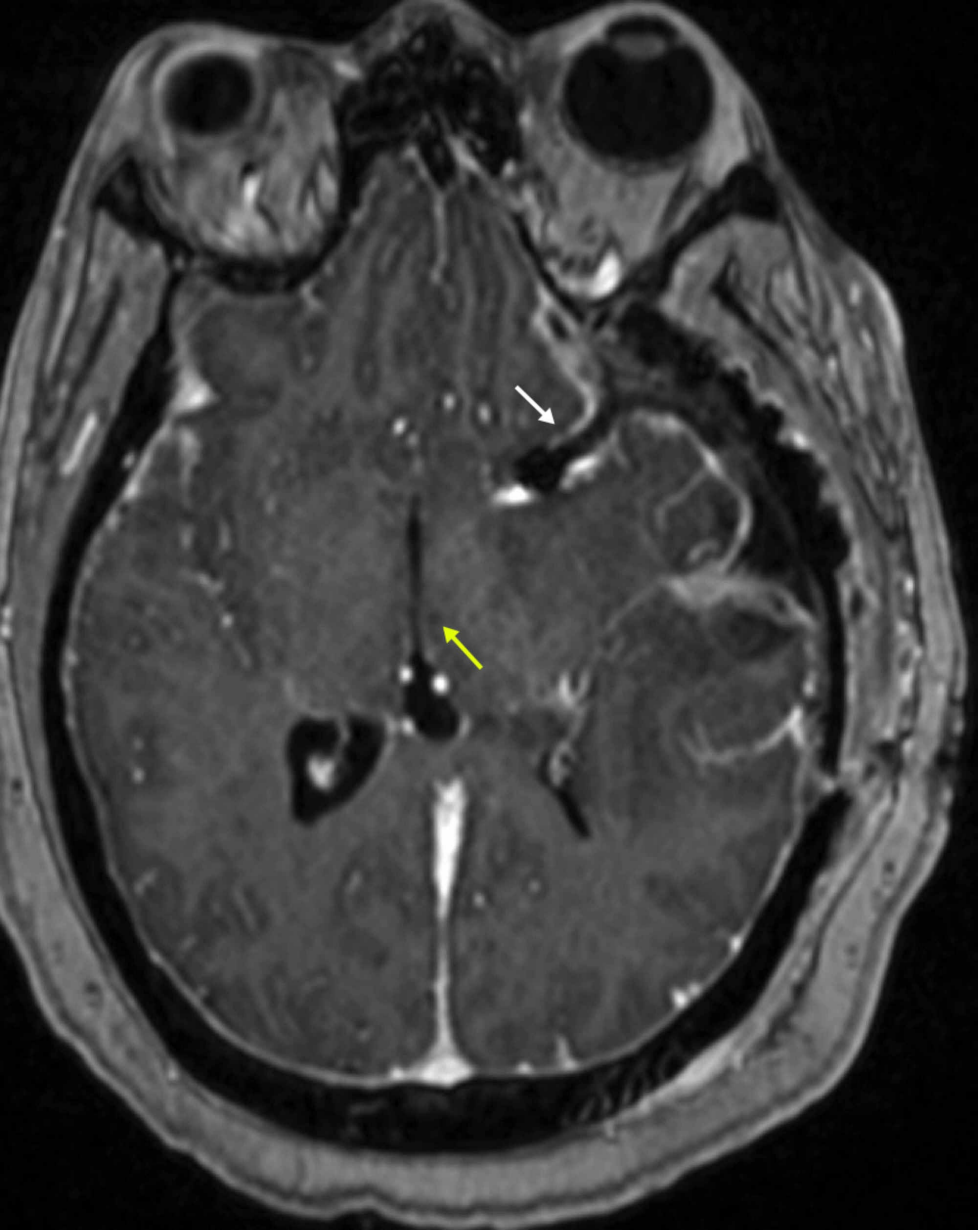
Postoperative MRI brain with contrast (axial view) shows the tumor resection bed (white arrow) and the thalamic punctate infarct (yellow arrow).

Postoperatively, the patient developed transient mild right-sided hemiparesis (4/5 muscle strength grade) that completely resolved by postoperative day 3. Postoperative cerebral angiography showed complete aneurysm occlusion (Figure 3), and brain magnetic resonance imaging showed gross total resection of the tumor with a punctate infarct in the left anterior thalamus (Figure 4). The final histopathological analysis of the tumor revealed a diagnosis of small cell carcinoma of the lung. The patient was discharged to a rehabilitation facility, and she was subsequently treated with stereotactic radiosurgery of the tumor resection cavity. At four-month follow-up, she remained neurologically intact and was undergoing chemotherapy for lung cancer diagnosis. 

## Discussion

The coexistence of brain tumors and unruptured intracranial aneurysms is an infrequent clinical scenario, and it poses a unique challenge to neurosurgeons. The probability of association between brain tumors and unruptured aneurysms is approximately 2.3%-7.7%, but it appears to vary by tumor type. Specifically, the coincidence of intracranial meningiomas and unruptured aneurysms has been reported to be about 1% compared to 3.9% for pituitary adenomas or craniopharyngiomas and unruptured aneurysms [6-10]. Manara et al. reported that 17% of patients with acromegaly had incidental aneurysms [11].

Multiple theories have been posited regarding the relationship between coexisting intracranial neoplasms and aneurysms. Pia et al. hypothesized that increased regional blood flow to the tumor can promote aneurysm formation due to increased hemodynamic stress on the adjacent arteries. The report also suggested that dysgenetic factors could contribute to the development of both lesions [12]. Some authors also suggested that local factors, such as tumor adhesion to the arterial wall and subsequent injury to the adventitia, can induce aneurysm development. There have also been studies showing a positive correlation between the serum level of growth hormone and aneurysm formation [11,13].

There is no consensus on the optimal management strategy for coexisting brain tumors and unruptured aneurysms. Our understanding of the behavior of untreated aneurysms during the perioperative period is limited. Aneurysm rupture has been reported during the follow-up course of brain tumor treatment with resection, stereotactic radiosurgery, or medical therapy [1,3,5]. The premise for this phenomenon in the case of tumor resection is destabilization of the aneurysm wall by the local hemodynamic changes and systemic stress response triggered by treatment. Due to the sparse literature pertaining to the relationship between these two entities, it remains unclear whether one should use the same rupture risk stratification and treatment paradigms for isolated unruptured aneurysms as those coexisting with brain tumors. Additionally, one must consider tumor pathology and location in the combined management strategy of both lesions.

In the present case, we decided to resect the tumor to obtain tissue for diagnosis and to relieve mass effect. Since the tumor was dural-based with avid contrast enhancement, we performed a preoperative catheter cerebral angiogram and transarterial embolization, which revealed an incidental left ACA proximal A1 segment aneurysm. Natural history studies of unruptured aneurysms have reported a very low rupture risk for small (<5 mm in maximum diameter) anterior circulation aneurysms [14-18]. However, ACA aneurysms arising from the proximal A1 segment are atypical, as they present in relatively younger patients (mean age 43.5 years) and with a smaller size than aneurysms in the most frequent locations. These rare aneurysms also appear to rupture at a smaller size (mean rupture size 3.3 ± 0.9 mm) than their more common counterparts [19,20]. The unusual and potentially higher risk location influenced our decision to treat the ACA proximal A1 aneurysm in our patient 

Several reports have documented surgical or endovascular treatment of aneurysms coexisting with brain tumors. There appeared to be a trend toward contemporaneous surgical aneurysm clipping and tumor resection when feasible, whereas endovascular therapy or observation was preferred when the aneurysm was distant or contralateral to the tumor [1,3,4,6]. We favored aneurysm treatment with surgical clipping because the aneurysm was ipsilateral to the tumor and the operative corridor could be obtained through the same craniotomy [19].

## Conclusions

The coexistence of intracranial aneurysms and brain tumors is uncommon, and the mechanisms for this association remain elusive. Patients with these dual diagnoses can be challenging to successfully manage. In cases for which treatment of each lesion is indicated and both can be safely accessed from a single operative approach or craniotomy, concurrent surgical intervention for the tumor and aneurysm is justifiable in appropriately selected cases. Since no consensus regarding the optimal treatment algorithm for coincident intracranial neoplasms and aneurysms exists, an individualized strategy accounting for tumor, aneurysm, and patient characteristics is necessary.

## References

[1] Akutsu N, Hosoda K, Ohta K, Tanaka H, Taniguchi M, Kohmura E (2014). Subarachnoid hemorrhage due to rupture of an intracavernous carotid artery aneurysm coexisting with a prolactinoma under cabergoline treatment. J Neurol Surg Rep.

[2] Drazin D, Spitler K, Cekic M (2013). Incidental finding of tumor while investigating subarachnoid hemorrhage: ethical considerations and practical strategies. Sci Eng Ethics.

[3] Hoya K, Yoshimoto Y, Shin M, Nemoto S (2011). Rupture of an internal carotid artery aneurysm within a clinoidal meningioma following stereotactic radiosurgery. Acta Neurochir.

[4] Park KY, Kim BM, Kim DJ (2016). Preoperative coiling of coexisting intracranial aneurysm and subsequent brain tumor surgery. Korean J Radiol.

[5] Rustagi T, Uy EM, Rai M, Kannan S, Senatus P (2011). Intracranial hemorrhage from undetected aneurysmal rupture complicating transphenoidal pituitary adenoma resection. Conn Med.

[6] Spitler K, Drazin D, Hanna G, Patel A, Chu R (2013). Association of intracranial aneurysms with meningiomas, pituitary adenomas, and gliomas: review of possible interrelationships. ISRN Neurol.

[7] Fischer BR, Palkovic S, Holling M, Niederstadt T, Jeibmann A, Wassmann H (2009). Coexistence of cerebral aneurysm and meningioma--pure accident?. Clin Neurol Neurosurg.

[8] Kim YH, Lee YJ, Han JH (2015). Association of intracranial aneurysms and meningiomas: a case-control study. J Neurosurg.

[9] Oh MC, Kim EH, Kim SH (2012). Coexistence of intracranial aneurysm in 800 patients with surgically confirmed pituitary adenoma. J Neurosurg.

[10] Oshino S, Nishino A, Suzuki T (2013). Prevalence of cerebral aneurysm in patients with acromegaly. Pituitary.

[11] Manara R, Maffei P, Citton V (2011). Increased rate of intracranial saccular aneurysms in acromegaly: an MR angiography study and review of the literature. J Clin Endocrinol Metab.

[12] Pia HW, Obrador S, Martin JG (1972). Association of brain tumours and arterial intracranial aneurysms. Acta Neurochir.

[13] Kandel E, Ludkovskaya I, Dobjansky N (1986). Aneurysm inside meningioma. Case report. Acta Neurochir.

[14] Juvela S, Porras M, Poussa K (2000). Natural history of unruptured intracranial aneurysms: probability of and risk factors for aneurysm rupture. J Neurosurg.

[15] Lee JM, Joo SP, Kim TS, Go EJ, Choi HY, Seo BR (2010). Surgical management of anterior cerebral artery aneurysms of the proximal (A1) segment. World Neurosurg.

[16] Park HS, Choi JH, Kang M, Huh JT (2013). Management of aneurysms of the proximal (A1) segment of the anterior cerebral artery. J Cerebrovasc Endovasc Neurosurg.

[17] Wiebers DO, Whisnant JP, Huston J (2003). Unruptured intracranial aneurysms: natural history, clinical outcome, and risks of surgical and endovascular treatment. Lancet.

[18] Greving JP, Wermer MJ, Brown RD (2014). Development of the PHASES score for prediction of risk of rupture of intracranial aneurysms: a pooled analysis of six prospective cohort studies. Lancet Neurol.

[19] Jang CK, Jang EW, Cho KC (2018). Radiographic and microsurgical characteristics of proximal (A1) segment aneurysms of the anterior cerebral artery. Neurol Sci.

[20] Cho YD, Ahn JH, Jung SC (2014). Coil embolization in precommunicating (A1) segment aneurysms of anterior cerebral artery. Neuroradiology.

